# Exoproduction and Biochemical Characterization of a Novel Serine Protease from *Ornithinibacillus caprae* L9^T^ with Hide-Dehairing Activity

**DOI:** 10.4014/jmb.2108.08037

**Published:** 2021-11-20

**Authors:** Xiaoguang Li, Qian Zhang, Longzhan Gan, Guangyang Jiang, Yongqiang Tian, Bi Shi

**Affiliations:** 1 Key Laboratory of Leather Chemistry and Engineering, Ministry of Education and College of Biomass Science and Engineering, Sichuan University, Chengdu 610065, P.R. China; 2 Key Laboratory of Bio-Resources and Eco-Environment, Ministry of Education and College of Life Sciences, Sichuan University, Chengdu 610065, P.R. China

**Keywords:** Characterization, *Ornithinibacillus caprae*, optimization, serine protease, dehairing

## Abstract

This study is the first report on production and characterization of the enzyme from an *Ornithinibacillus* species. A 4.2-fold increase in the extracellular protease (called L9^T^) production from *Ornithinibacillus caprae* L9^T^ was achieved through the one-factor-at-a-time approach and response surface methodological optimization. L9^T^ protease exhibited a unique protein band with a mass of 25.9 kDa upon sodium dodecyl sulfate-polyacrylamide gel electrophoresis. This novel protease was active over a range of pH (4–13), temperatures (30–80°C) and salt concentrations (0–220 g/l), with the maximal activity observed at pH 7, 70°C and 20 g/l NaCl. Proteolytic activity was upgraded in the presence of Ag^+^, Ca^2+^ and Sr^2+^, but was totally suppressed by 5 mM phenylmethylsulfonyl fluoride, which suggests that this enzyme belongs to the serine protease family. L9^T^ protease was resistant to certain common organic solvents and surfactants; particularly, 5 mM Tween 20 and Tween 80 improved the activity by 63 and 15%, respectively. More importantly, L9^T^ protease was found to be effective in dehairing of goatskins, cowhides and rabbit-skins without damaging the collagen fibers. These properties confirm the feasibility of L9^T^ protease in industrial applications, especially in leather processing.

## Introduction

According to the China Leather Industry Association (www.chinaleather.org), China is one of the most active and promising leather trade markets in the world, with a genuine leather production of about 529 million square meters in 2019. The leather industry prevailingly uses skins, by-products of livestock, as raw materials for processing, involving many physical and chemical treatments such as soaking, degreasing, dehairing, pickling and tanning. Among these processes, the dehairing of animal hides is considered to be the most polluting [1]. The traditional dehairing method requires various toxic chemical reagents such as Na_2_S, NaHS and CaCl_2_ [2], which can cause serious environmental pollution through the discharge of toxic gases and solid waste [3, 4]. In order to overcome the hazards caused by chemicals, microbial proteases have been proposed as green alternatives [5].

Proteases represent a large and diverse group of hydrolytic enzymes involved in breaking down chains of amino acids. The peptide chains are repeatedly folded or supercurled to form a three-dimensional structure with active pockets or crevices. The shape of the catalytic pockets depends on the arrangement of amino acid residues, and only substrates of a certain size and shape can fit and bind to them. Thus, protease can efficiently cleave the peptide bond to break down protein into smaller and simpler mixtures [6]. In view of their specifity, high efficiency, and environmental friendliness, proteases are regarded as important industrial biocatalysts, which has caused an upsurge in the exploitation of protease-producing microorganisms [7]. To date, a large number of extracellular proteases have been reported and characterized from various microorganisms, including *Paecilomyces marquandii* MZKI B639 [8], *Bacillus subtilis* BLBc 11 [9], *Lactobacillus curvatus* R5 [10], *Nocardiopsis dassonvillei* OK-18 [11] and *Streptomyces koyangensis* TN650 [12]. It is worth noting that most of the reported extracellular proteases are serine proteases [13, 14], which display a broad range of biotechnological applications, including food production [15], feather degradation [16], stain washing [17] and skin dehairing [14]. As candidates for enzymatic dehairing, serine proteases can specifically attack only certain proteinaceous substances and the epidermis without damaging the skin collagen since they often lack collagenase activity [14, 18, 19]. This allows for the ability to retain the inherent collagen components in the dermis and recover high-quality leather while also reducing contaminants in wastewater.

Over the past decades, the reported extracellular serine proteases have been principally derived from *Bacillus* strains; however, no attention has been paid to the rare species of the genus *Ornithinibacillus*. At the time of writing, the genus *Ornithinibacillus* comprises 12 species with validly published names [20], and its members are usually aerobic moderate halophiles. Despite these studies, no report exists on the enzymes from *Ornithinibacillus* strains, as far as the authors know. Thus, further studies on enzymes produced by *Ornithinibacillus* strains are highly desirable in order to expand the existing toolbox of industrial enzymes and to realize potential applications. Previous work scientifically classified an extracellular protease-producing strain as *Ornithinibacillus caprae* L9^T^ [21]. In view of the above, in this study, we adopted effective optimization strategies to improve the productivity of *O. caprae* L9^T^ protease. Meanwhile, the biochemical and molecular characteristics of the protease sample were studied. Further, the crude enzyme was used as a biocatalyst for the dehairing of animal hides to estimate its application potential in the leather industry.

## Materials and Methods

### Microorganism and Reagents

*O. caprae* L9^T^, a moderately halophilic bacterium isolated from a goat hide [21], was deposited in the Korean Collection for Type Cultures (KCTC) and the laboratory of industrial biotechnology with the accession numbers KCTC 43176 and L9, respectively. Unless otherwise stated, all chemical reagents used in this work are analytically pure and obtained from several commercial companies, including Sangon Biotech (China), Sigma-Aldrich (USA), and Takara (Japan).

### Genomic Analysis

The assembled genome of strain L9^T^ was submitted to GenBank with the accession number WOCA00000000. The phylogenetic tree of the genomes of strain L9^T^ and its related taxa was reconstructed using the Type Strain Genome Server (TYGS) [22]. Simultaneously, in order to assess the genetic diversity of strain L9^T^, the DNA sequences were performed with BLASTX search against the non-redundant (NR) protein database [23].

### Protease Activity Assay

The protease activity was determined according to the procedure GB/T 23527-2009 [24] stated by the National Standardization Administration of China (SAC) with slight modifications. The appropriately diluted enzyme solution (0.2 ml) was mixed with 0.2 ml of 20 g/l casein dissolved in Tris-HCl buffer (50 mM, pH 7). The mixture was then incubated for 10 min at 70°C, and the reaction was quenched by addition of 0.4 ml of 65.4 g/l trichloroacetic acid (TCA). After violent shaking, the precipitated proteins were eliminated by centrifugation at 12,000 ×*g* and 4°C for 5 min. The resultant supernatant (0.4 ml) was then mixed with 2 ml of 42.4 g/l Na_2_CO_3_ and 0.4 ml of 1 M Folin & Ciocalteu's phenol reagent. Later, the mixture was allowed to stand for 20 min at 40°C, and the absorbance was monitored at 680 nm against the control using a UV/Vis spectrophotometer. Additionally, a negative control was performed in the same way and the substrate was added after adding TCA. In consequence, one unit of proteolytic activity (U/ml) was defined as the amount of enzyme required to liberate 1 μg of tyrosine per minute under specified assay conditions.

### Optimization of Protease Production by Strain L9^T^

Strain L9^T^ was precultured for 12 h in Luria-Bertani broth supplemented with 100 g/l NaCl as a seed solution to inoculate (2%, v/v) the subsequent enzyme-producing fermentation medium. Thereafter, the effect of fermentation variables on the enzyme production of *O. caprae* L9^T^ was investigated using the one-variable-at-a-time approach and response surface methodology (RSM). The three independent variables (carbon source, nitrogen source and initial pH) that had the most significant impact on protease production were selected. Later, the optimum concentration and interaction of these three factors for enhancing the protease production was studied by Box-Behnken design (BBD). Each factor was studied at three different levels: -1, 0 and +1 (Table S1). A set of 17 experiments was generated by design expert software (Table S2), in which 5 experiments were repeated at the central level to evaluate the linear and curvature effects of the variables. Accordingly, the proteolytic activity was designated as a response, and the regression analysis, response surface model and significance tests were performed using DesignExpert software (Stat-Ease, Inc., USA).

### Partial Purification of Protease

*O. caprae* L9^T^ culture supernatant, containing secreted protease, was harvested through centrifugation at 8,000 ×*g* and 4°C for 5 min, and then dialyzed in a dialysis bag (8 kDa MWCO) against repeated changes of Tris-HCl buffer (pH 7). The dialyzed sample was prepared, and stored at 0°C for further analysis.

### SDS-PAGE, Zymography and Mass Spectrometry

The purity and molecular weight of the enzyme were determined by sodium dodecyl sulfate-polyacrylamide gel electrophoresis (SDS-PAGE) according to the method of Laemmli [25] under denaturing conditions. In the meantime, the proteolytic activity was confirmed by zymography according to the method described by Garciacarreno *et al*. [26] with slight changes. Briefly, the enzyme sample, mixed with an equal volume of 2×SDS non-reducing loading buffer, without boiling, was electrophoresed at constant current. First, after electrophoresis, the gel was immersed in Tris-HCl buffer (50 mM, pH 7) supplemented with 2.5% (v/v) Triton X-100 to eliminate SDS. Second, the gel was washed twice in Tris-HCl buffer for 40 min with agitation to remove Triton X-100. Third, the hydrolysis reaction occurred inside the gel during incubation with 10 g/l casein at 40°C for 12 h. Finally, the gel was stained with a dye solution consisting of 1 g/l Coomassie Brilliant Blue R-250, 8% (v/v) acetic acid and 25% (v/v) ethanol for 12 h at room temperature. In general, the presence of a white band on a dark blue background indicates the existence of protease activity.

Thereafter, the area of active enzyme was extracted from the electrophoresis gel for reduction, alkylation, trypsin digestion, and desalting. The mixture containing peptides of different sizes was detected using liquid chromatography-tandem mass spectrometry (LC-MS/MS) at Sangon Biotech. Importantly, ProteinPilot software (version 4.5) was employed to retrieve the obtained peptide sequences in the GenBank database.

### Biochemical Characterization

**Determination of temperature on L9^T^ protease activity and stability**. A range of various temperatures (30–80°C at 5°C intervals) was used to determine the optimum temperature, and the maximum enzyme activity was considered as 100% activity. Thermostability of L9^T^ protease was determined after incubation of the enzyme at 30–80°C for 1 h. The residual enzymatic activities were measured at the optimal temperature, and the enzyme that had not been incubated served as the 100% control.

**Effect of pH on L9^T^ protease activity and stability**. The fermentation supernatant was dialyzed against Tris-HCl buffer (5 mM, pH 7) using an 8 kDa dialysis bag at 4°C for 24 h and the buffer was changed at 4 h intervals. Then, the dialyzed enzyme was diluted in several different pH buffers (pH 2–13) in order to estimate the impact of pH on proteolytic activity. Maximum enzyme activity for the buffer solution was considered as 100%. Five buffer systems (50 mM) including glycine-HCl (pH 2–4), CH_3_COONa-CH_3_COOH (pH 5–6), Tris-HCl (pH 7–9), glycine-NaOH (pH 10–11), and KCl-NaOH (pH 12–13) were used. Similarly, the pH stability was evaluated by preincubating the dialyzed enzyme in different pH buffers at 25°C for 1 h, and residual enzymatic activity was measured at pH 7 and 70°C. The unincubated enzyme activity measured with Tris-HCl buffer (50 mM, pH 7) was considered as a control (100%).

**Effect of NaCl on L9^T^ protease activity and stability**. To investigate the effect of NaCl on proteolytic activity, the assay was performed at 70°C with L9^T^ protease in the presence of varying salt concentrations (0–220 g/l). As for its salt tolerance, L9^T^ protease was pre-incubated in different salinities (0–220 g/l with an interval of 20 g/l) for 1 h prior to determination of remaining activities. The activity of L9^T^ protease in the absence of NaCl was calculated as 100%.

**Effect of chemical agents on protease activity**. The effects of various chemicals on protease catalysis were studied by pre-incubating L9^T^ protease for 1 h with each reagent. Dimethyl sulfoxide (DMSO), H_2_O_2_ and nonionic surfactants, including Tween 20, Tween 80 and Triton X-100, were provided at the working concentration of 1%(v/v). Similarly, organic solvents, including acetone, benzene, ethanediol, ethanol, glycerol, n-hexane, isopropanol and methanol were evaluated at 5 and 10% (v/v), respectively. Other types of chemicals were used at a final concentration of 5 mM; reducing agents: dithiothreitol and β-mercaptoethanol; protease inhibitors: ethylene-diamine-tetraacetic acid (EDTA); phenylmethylsulfonyl fluoride (PMSF) and ethylene glycol-bis (β-aminoethyl ether)-N,N,N’,N’-tetraacetic acid (EGTA). The corresponding remaining activities were determined under the standard conditions, and the enzyme solution without any additives was set as a control.

**Effect of different metal ions on protease activity**. The effects of various monovalent (Li^+^, K^+^ and Ag^+^), divalent (Mg^2+^, Ca^2+^, Mn^2+^, Fe^2+^, Co^2+^, Cu^2+^, Zn^2+^, Sr^2+^ and Ba^2+^) and trivalent (Cr^3+^ and Fe^3+^) metal ions (5 mM) on protease stability were investigated by pre-incubating L9^T^ protease for 1 h with each metal cation. The remaining activities were measured at pH 7 and 70°C, and the enzyme activity in the absence of any metal ions was considered 100%.

**Substrate specificity.** The hydrolysis capacities of L9^T^ protease towards different protein substrates including azocasein, bovine serum albumin (BSA), casein, collagen and keratin were evaluated. All substrates were prepared with 50 mM Tris-HCl buffer (pH 7) at a final concentration of 20 g/l. Enzymatic activities were measured on each substrate according to standard conditions. The maximum activity was expressed as 100%, corresponding to the best substrate.

### Dehairing of Animal Hides

Fresh cowhides, goatskins and rabbit skins were purchased from local farms, and permission was obtained from farmers to use these animal skins for experiments. The obtained animal hides were divided into square shapes of approximately 6 cm × 6 cm by a sharp knife. The small squares of hairy animal skin were rinsed with tap water to remove blood, mud, and insoluble impurities, and then drained at room temperature. Most of the squares were separately placed into flasks containing 100 ml of diluted enzyme solution (600 U) and 3% (w/w) Na_2_S, while the rest was soaked in 100 ml of Tris-HCl buffer (50 mM, pH 7) as a control. Later, all flasks were placed in a constant temperature shaker (38°C and 150 rpm) for 24 h. After incubation, the skins were taken out, and the potential of L9^T^ protease for application in the leather industry was appraised by dehairing efficacy and histological examination.

Samples of approximately 1 cm^2^ in size were sliced from the goatskins and treated with L9^T^ protease, Na_2_S, and Tris-HCl buffer. The samples were thoroughly washed and fixed with 40 g/l paraformaldehyde at indoor temperature for 24 h. After dehydration with ethanol, the samples were embedded in paraffin block and cut into slices of 3 μm by a microtome. The slices were stained using Masson’s trichrome staining and hematoxylin and eosin (HE), and then the stained slices were observed under a microscope.

### Statistical Analysis

All determinations were done in three independent replicates, and the control experiments were carried out under the same conditions. All data were analyzed using OriginPro software (version 8.5), and the results were expressed as mean ± standard deviation (SD).

## Results and Discussion

### Genomic Features

**Fig. 1:**
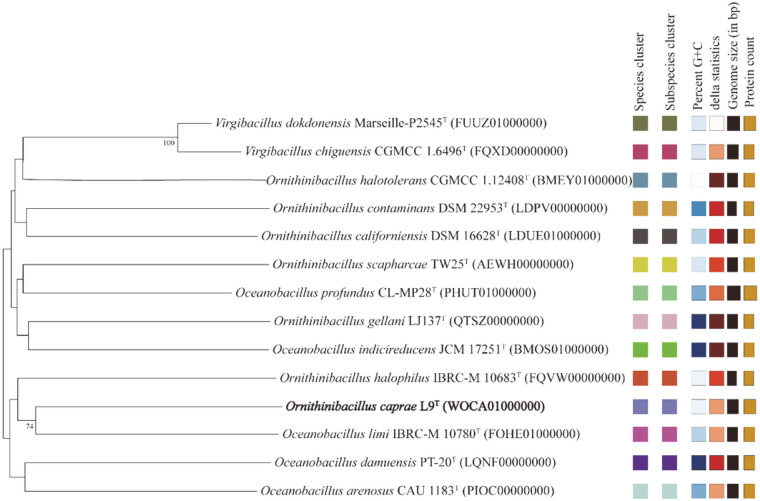
Phylogenomic tree based on TYGS results showing the relationship between *O. caprae* L9^T^ and its related type strains. The numbers above branches are the genome blast distance phylogeny pseudo-bootstrap support values > 70% from 100 replications, with an average branch support of 90.6%. Accession numbers are given in parentheses.

The family *Bacillaceae* was first proposed by Fischer and currently contains more than 100 recognized genera [20], including *Ornithinibacillus*, *Oceanobacillus* and *Virgibacillus*, which are close neighbors in terms of phylogenetic evolution. As depicted in Fig. 1, the species of the genera *Ornithinibacillus* and *Oceanobacillus* are intertwined, suggesting that their genomes contain some identical sequences or are highly similar. In the literature, some *Oceanobacillus* strains have been reported to produce protease [27-29], amylase, lipase and carboxymethyl cellulase [30, 31]. Thus, *Ornithinibacillus* species, the twin brother of *Oceanobacillus*, may also have the ability to secrete enzymes. Additionally, the 3944 coding DNA sequences of strain L9^T^ obtained by homology analysis were annotated by the NR database. The annotation results revealed that strain L9^T^ contains at least 25 genes capable of encoding serine protease, glycosidase, amylase, metalloprotease and lipase (Table S3). The diversity of the enzyme genes provides direction and theoretical support for subsequent studies. More excitingly, the hydrolytic circles formed by strain L9^T^ were clearly observed on milk agar plates, and the protease activity of 60.97 U/ml was detected in tryptic soytone broth (TSB) containing high amounts of NaCl.

### Statistical Optimization of Protease Production

**Response surface methodology for optimizing experimental design.** According to the single-factor experimental results (shown in the Supplementary Material), three significant independent variables, including yeast extract (A), urea (B) and initial pH (C), were selected to determine the optimum level of protease production. Based on the response values of 17 experiments listed in Table S2, a quadratic model for predicting the maximum protease yield (Y) was generated and presented as follows:

Y (U/ml) = 246.15–12.97A–32.89B–7.51C+0.35AB–37.30AC–8.68BC–42.35A^2^–68.29B^2^–77.57C^2^.

Table 1 summarizes the variance analysis results of the regression model. The results indicated that the model is significant since it has a high model *F*-value (= 107.4) and a very low probability value (< 0.0001). Besides, the input variables (A, B, C) and their interaction effects (AC, A^2^, B^2^ and C^2^) were also significant (*p*-value less than 0.05). Adequate precision measures the ratio of signal to noise, and a ratio > 4 is a necessary prerequisite for a good fit of the model. A ratio of 28.94 obtained from this study demonstrates that the model can be used to navigate the design space. More importantly, most of the variability in the test data was explained by the model, with the correlation coefficient (R^2^) of 99.28% and adjusted R^2^ of 98.36%. These collective results further confirm the feasibility of this model to predict the production of L9^T^ protease.

**Fig. 2:**
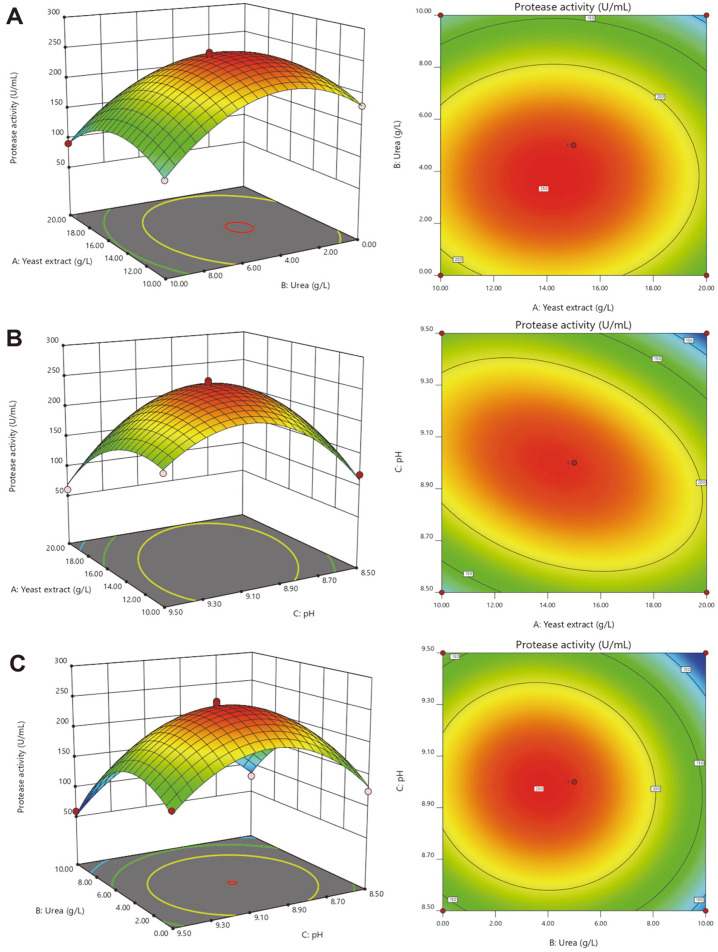
Three-dimensional response surface graphs and contour plots of extracellular protease production by *O. caprae* L9^T^ elucidating the interaction between: (A) yeast extract and urea; (B) yeast extract and pH; (C) urea and pH.

The interaction effects and optimum values of a combination of the three independent factors for maximum protease production by *O. caprae* L9^T^ were represented by three-dimensional response surface graphs and contour plots (Fig. 2). According to Figs. 2A and 2B, the proteolytic activity first increased and then decreased with increasing levels of yeast extract in the fermentation medium. It can also be seen from Fig. 2B that there is a significant interaction between yeast extract and pH since the contour is elliptical in shape.

**Validation of the optimized process conditions**. Through analysis of the regression equation, the model predicted that when yeast extract, urea and initial pH were set to 14.3 g/l, 3.8 g/l, and 9, respectively, maximum L9^T^ protease yield (251.12 U/ml) can be achieved. Then, three repeated experiments were executed under the predicted optimal conditions. The experimental L9^T^ protease yield was 255.86 ± 0.71 U/ml, which was comparable to the predicted one, confirming the model’s authenticity. After statistical optimization, the production of L9^T^ protease increased by 319.65% when compared with that obtained under the original medium and unoptimized fermentation conditions (60.97 U/ml).

### Gel Electrophoresis and Molecular Analysis

**Fig. 3:**
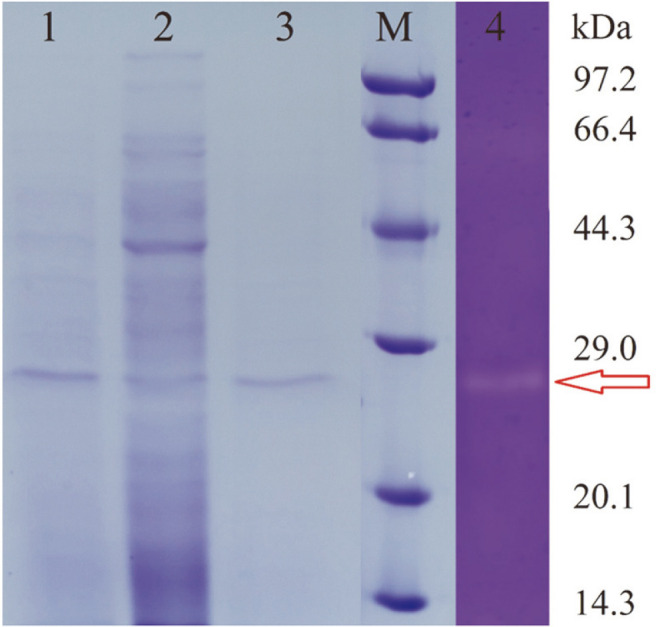
SDS-PAGE analysis of the extracellular protease from *O. caprae* L9^T^. Lane 1: fermentation broth; lane 2: bacterial cell; lane 3: L9^T^ protease; lane M: standard protein marker (kDa); lane 4: zymogram of protease with casein.

As shown in Fig. 3, a single protein band with molecular mass ranging from 20.1–29 kDa was obtained with the enzyme preparation. Zymogram activity staining showed the proteolytic activity as a white band against the blue background of the gel, as a result of the casein hydrolysis. Furthermore, the results from MS analysis confirmed that L9^T^ protease had multiple peptides with amino acid sequences of TGEEIDKRVTPFSIIG or KIRRHFTN. BLAST result then revealed that these peptides were affiliated to the protein WP_155668210.1 (333 amino acids) of *O. caprae* L9^T^. Based on the NCBI conserved domain analysis, WP_155668210.1 was identified to be a serine protease containing signal peptide (1-24 amino acids), intervening propeptide (25-97 amino acids) and mature peptide (98-333 amino acids). Thus, it is speculated that L9^T^ protease is the active mature peptide of protein WP_155668210.1, with a molecular weight of 25.9 kDa. In correlation with present study, a surfactant-stable serine protease from *Bacillus* sp. B001 showed a molecular mass of 28 kDa [32]. Similarly a detergent stable alkaline serine protease named BM1 with a mass of 29 kDa was also reported from *Bacillus mojavensis* A21 [17].

### Characterization of L9^T^ Protease

**Fig. 4:**
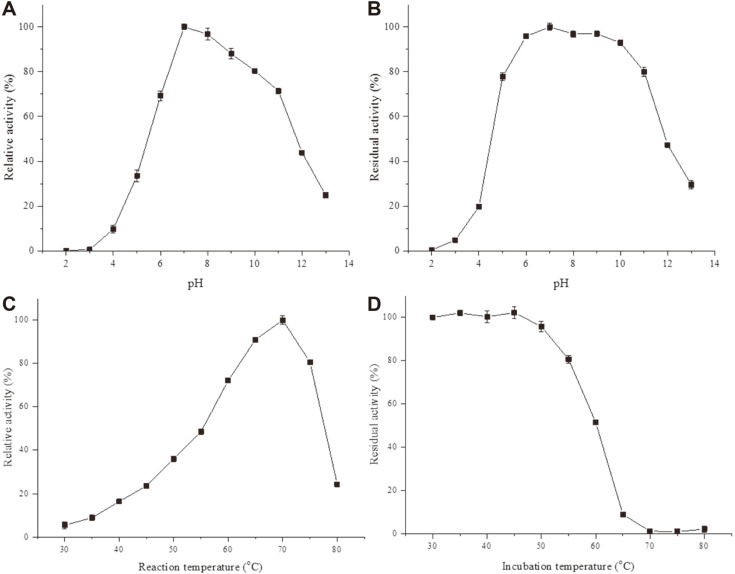
pH and temperature profile of the protease from *O. caprae* L9^T^. Effects of pH on the activity (**A**) and stability (**B**) of L9^T^ protease. (**C**) The activity of L9^T^ protease at various reaction temperatures (30–80°C at 5°C intervals) was determined in Tris-HCl buffer (pH 7). (**D**) Thermal stability was studied by pre-incubating L9^T^ protease at 30–80°C temperatures for 1 h, activity without pre-incubation was taken as 100%. Reported results are the average of three independent experiments with the standard deviation presented as error bars.

**Effect of pH, temperature and NaCl on enzymatic activity and stability**. The partially purified L9^T^ protease displayed proteolytic activity within a broad pH range of 4–13 (Fig. 4A), with the maximal activity at a pH of 7. The pH action of L9^T^ protease is lower than that of some reported serine proteases, including STAP (pH 2–13) from *S. koyangensis* TN650 [12] and KERUS (pH 2–13) from *Brevibacillus brevis* US575 [14], but higher than that of AprB (pH 5–13) from *Bacillus* sp. B001 [32]. In addition, the pH stability profile (Fig. 4B) manifestly showed that L9^T^ protease was considerably stable in the pH range of 6 to 11, maintaining greater than 80% of original activity after incubation for 1 h. These properties make L9^T^ protease a good candidate for industrial applications such as use in detergents [33], tanning processes [3] or as a biocontrol agent [34].

The proteolytic activity was recognizable over a wide range of temperatures (30 to 80°C) with optimum activity at 70°C as shown in Fig. 4C. The findings are consistent with the enzyme from *N. dassonvillei* OK-18 reported by Sharma *et al*. [11], but higher than protease BM2 produced by *B. mojavensis* A21 [17], which presented an ideal temperature of 60°C. Obviously, L9^T^ protease was highly stable at a temperature range of 30 to 45°C as revealed by the thermal stability profile (Fig. 4D). However, the protease activity decreased to 51.45% of the initial activity after 1 h of incubation at 60°C, and completely vanished after 1 h of incubation at 70–80°C. Earlier, the serine keratinase of *Brevibacillus parabrevis* CGMCC 10798 showed about 90% loss of its original activity upon exposure to 70°C for 1 h.

**Fig. 5:**
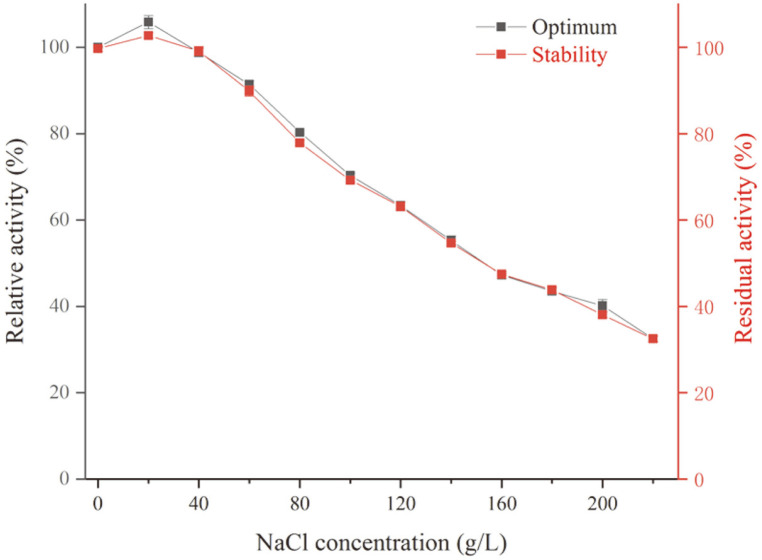
Effect of various concentrations of NaCl on activity and stability of L9^T^ protease. The enzymatic activity was determined at pH 7 and 70°C using casein as the substrate. The enzyme activity of a control (without any NaCl), incubated under similar conditions, was taken as 100%. Each value represents the mean of three replicates, and ± standard errors are reported.

The effect of NaCl on protease activity was measured in the salinity ranging from 0–220 g/l at pH 7 and 70°C. As shown in Fig. 5, L9^T^ protease was active at salinity from 0 to 220 g/l and had an optimum at 20 g/l. Similarly, strains like *Bacillus iranensis* X5B [6] and *Bacillus* sp. NPST-AK15 [33] were observed to produce serine protease which had optimum NaCl concentrations of about 57 and 15 g/l, respectively. It was also observed that L9^T^ protease retained 80.31, 55.23, and 32.49% of its activity at NaCl concentrations of 80, 140, and 220 g/l, respectively, indicating a certain resistance of the protease to high salinity stresses. Notably, salinity stability showed that this protease was stable at all tested NaCl concentrations after 1 h of incubation. Similar results were also reported for the alkaline protease from *Bacillus* sp. NPST-AK15, where the enzyme showed high stability in the range of 0–200 g/l NaCl. From the above results, L9^T^ protease is confirmed as a slightly halophilic enzyme, which may be useful for certain biotechnological processes that depend on salinity.

**Effect of various metal ions on L9^T^ protease activity**. The effects of various metal ions on the activity of L9^T^ protease are summarized in Table 2. In the presence of 5 mM Ag^+^, Ca^2+^, and Sr^2+^, the proteolytic activity significantly increased to 104.46, 104.40, and 120.37%, respectively in comparison to the control. Indeed, calcium ions are known to improve activity and prevent conformational changes in many serine proteases [12, 14, 16]. In the same manner, the activity of a metallo-serine keratinase from *Streptomyces aureofaciens* K13 was enhanced to 109.54% in the presence of 5 mM Sr^2+^ [35]. These observations may be explained by the fact that (i) the spatial structure of the protease contains several Sr^2+^ binding sites, where Sr^2+^ may act as a salt or ion bridge to maintain the structure conformation of the enzyme or to stabilize the binding of the substrate and enzyme complex [36]; (ii) Sr^2+^ probably enhances the binding affinity of the substrate (casein) to the active site of protease [37]. In addition, the activation effect of Sr^2+^ on L9^T^ protease is stronger than that of Ca^2+^ and Ag^+^, implying that Sr^2+^ is the best inducer of this enzyme at a certain concentration. The concentration of 5 mM of the ions Li^+^, K^+^, Ba^2+^, Mg^2+^, Mn^2+^, and Co^2+^ resulted in little effect on L9^T^ protease, while in Fe^2+^, Cu^2+^, Zn^2+^, and Cr^3+^ ions there was a moderate inhibition of activity. Similar results were found by Patil and Chaudhari [38], who also observed a slight effect of the metal ions K^+^, Mn^2+^, and Mg^2+^ on the reduction of proteolytic activity (9.2, 2.0, and 3.5%, respectively), as well as severe inhibition of this activity by Cu^2+^ and Zn^2+^. However, Fe^3+^ strongly inhibited the protease activity, recording 28.55% of the control activity, which may be due to the fact that Fe^3+^ readily captures electrons from the enzyme surface through strong oxidation, thus destabilizing the enzyme.

**Effect of chemicals on protease activity.** The impacts of different organic solvents on L9^T^ protease were evaluated to inspect its further potential application. As shown in Table 3, L9^T^ protease was quite stable in the solvents tested, especially at a concentration of 5% (v/v). More interestingly, the protease also exhibited high stability after 1 h incubation with 10% (v/v) benzene, glycerol and n-hexane, with residual activities of 100.76, 99.50, and 95.70%, respectively. A similar stability of serine protease HAOP in benzene and hexane has been reported [39]. However, increasing concentrations of ethanol, isopropanol and methanol caused a gradual decline of L9^T^ protease activity, which is in accord with some other serine proteases [11, 40]. In the present study, the remarkable stability of L9^T^ protease in the presence of common organic solvents makes it interesting for the synthesis of peptides and esters.

At the same time, the effects of surfactants on the activity of L9^T^ protease were appraised, and the corresponding results are listed in Table 4. L9^T^ protease showed excellent stability and compatibility with some nonionic surfactants. In fact, the protease activities were significantly enhanced by Tween 20 and Tween 80, with enzyme activity rates of 163.41 and 115.01% of the control, respectively, at a concentration of 1% (v/v). However, the stimulatory effects of Tweens on serine proteases were not observed by Haddar *et al*. [17], Suwannaphan *et al*. [40] and Gegeckas *et al*. [41]. Moreover, 1% (v/v) of Triton X-100 inhibited the enzyme activity by 10% after 1 h of incubation, which is also observed with the alkaline metalloprotease (7.5% reduction) from *Pseudomonas aeruginosa* MTCC 7926 [38] and serine proteases (18% reduction) from *N. dassonvillei* OK-18 [11] and *Bacillus* sp. C4 SS-2013 [40], while in contrast to the keratinase (increase by 38.16%) derived from *S. aureofaciens* K13 [35]. L9^T^ protease was preincubated with different concentrations of DMSO (1, 5 and 10%, v/v) but no constraint was observed, and the enzyme retained 99.94% activity even after 1 h incubation with 10% (v/v) concentration. Conversely, the strong anionic surfactant SDS at 10 g/l inhibited the protease activity by about 77%, which is in agreement with most previously reported findings of enzyme denaturation and inactivation by SDS [33, 40]. Furthermore, H_2_O_2_ slightly lessened the protease activity by 11.82%. All these outstanding features ensure that L9^T^ protease is compatible with certain detergent formulations.

As seen in Table 4, the activity of L9^T^ protease was not markedly affected by 5 mM thiol reagents, including dithiothreitol and β-mercaptoethanol, which may be explained by the fact that sulfhydryl does not directly participate in the catalytic reaction [42]. The results signify that L9^T^ protease is a thiol-independent enzyme, similar to reported serine protease KERAB [43] and keratinolytic protease KERDZ [42]. The protease retained 30.44 and 49.85% of original activity in the presence of 5 mM chelators EDTA and EGTA, respectively, indicating that L9^T^ protease is not a metalloprotease but only uses certain cations as stabilizers [32, 44]. More importantly, L9^T^ protease was thoroughly suppressed by PMSF, suggesting that it belongs to the family of serine proteases [16].

**Substrate specificity profile of L9^T^ protease.** The activity of L9^T^ protease towards various substrates is presented in Fig. S4. As seen, the protease showed the highest activity toward casein, followed by azocasein, BSA and keratin. However, no activity was detected on gelatin and collagen. This finding supports the potential candidacy of L9^T^ protease as a biocatalyst for dehairing in the leather industry, as it lacks collagenase activity and does not damage skin collagen.

### Dehairing Performance of L9^T^ Protease

**Fig. 6:**
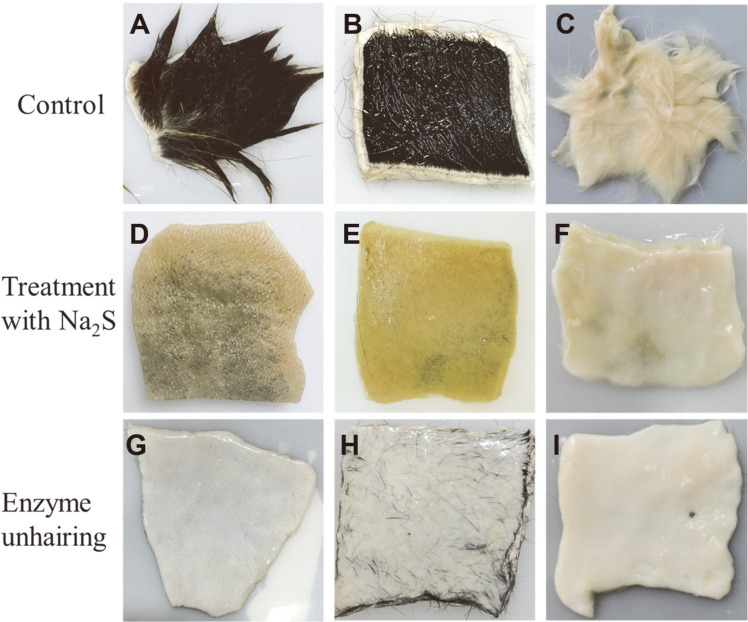
Dehairing performance of L9^T^ protease on various animal skins. Goatskins treated with Tris-HCl buffer (**A**), Na_2_S (**D**) and L9^T^ protease (**G**), respectively; cowhides treated with Tris-HCl buffer (**B**), Na_2_S (**E**) and L9^T^ protease (**H**), respectively; rabbit skins treated with Tris-HCl buffer (**C**), Na_2_S (**F**) and L9^T^ protease (**I**), respectively.

The dehairing efficacy of the extracellular enzyme from *O. caprae* L9^T^ on animal hides was assessed through organoleptic tests and histological analyses. As seen in Fig. 6, both Na_2_S and L9^T^ protease could effectively remove the hair from cowhides, goatskins and rabbit skins (Figs. 6D-6I); while the control hides, incubated under the same conditions, showed no sign of hair removal (Figs. 6A-6C). Obviously, the enzymatic dehaired pelts were white in color, and showed clean hair pores, with soft texture and smooth grain surface (Figs. 6G-6I); whereas, touch-visual tests revealed that the conventional dehaired pelts were not only yellow or dark brown in color, but also hard and wrinkled (Figs. 6D-6F).

In the traditional dehairing process, Na_2_S dissolved in water produces large amounts of hydroxide, which then destroys or even degrades the hair [45], resulting in the production of toxic gas H_2_S, suspended solids and highly alkaline wastewater. In contrast, as a bioactive catalyst, L9^T^ protease does not act directly on the hair shafts; instead, it could efficiently and specifically hydrolyze some proteinaceous substances in the hair pores, such as mucin, albumin, glycoprotein and globulin, thereby destroying the bond between the hair shaft and the hair follicle [45, 46]. The absence of these connections caused the hair to naturally detach from the skin under the condition of horizontal rotation without the need for other mechanical forces.

Histological studies on cross-sections of goatskins treated with Tris-HCl buffer, Na_2_S and L9^T^ protease using HE and Masson's trichrome staining are presented in Fig. S5. As shown, the epidermis of the goatskins was completely removed by treatment with Na_2_S and L9^T^ protease (Figs. S5b, S5c, S5e, S5f) compared to the blank control (Figs. S5a, S5d). The collagen fiber structure of the enzymatically dehaired pelt was more regular and intact (Figs. S5c, S5f) than that of the Na_2_S-treated pelt (Figs. S5b, S5e). In general, in Masson’s trichrome staining, collagen and non-collagenous substances appear blue and dark red, respectively. Thus, the results suggest that the enzymatic dehairing could well maintain the inherent collagen component in the dermal structure (Fig. S5f), while Na_2_S could damage some skin collagen (Fig. S5e).

In this study, L9^T^ protease could completely dehair goatskin and rabbit skin (Figs. 6G, 6I) in 24 h without any chemicals. However, it could not remove some short hairs on cowhide (Fig. 6H), probably because the thickness of cowhide hinders the penetration of enzymes and lacks mechanical pull. It is worth mentioning that microbial proteases with outstanding dehairing ability are stable in alkaline environment, especially between pH 8 and 10 [14, 47]. Hence, L9^T^ protease is noted as meeting this criterion and may be regarded as a promising candidate for dehairing in the leather industry.

In the current work, production, optimization, partial purification and characterization of a novel serine protease from *O. caprae* L9^T^ have been reported. Strain L9^T^ showed the highest protease production capacity (255.86 U/ml) after 72 h of fermentation at 37°C in the optimized medium containing 14.3 g yeast extract, 3.8 g urea, 130 g NaCl, 1 L distilled water and an initial pH of 9. L9^T^ protease appeared as a single band on SDS-PAGE gel with a molecular mass of 25.9 kDa. The optimal reaction pH and temperature of the protease were 7 and 70°C, respectively. Additionally, the protease was activated by 20 g/l NaCl and 5 mM metal ions, including Ag^+^, Ca^2+^ and Sr^2+^, and exhibited excellent compatibility with several nonionic surfactants and organic solvents. Further studies have shown that L9^T^ protease has a perceptible ability to dehair animal hides without any damage. These findings demonstrate that the protease secreted by *O. caprae* L9^T^ may be used for various industrial applications.

## Supplemental Materials

Supplementary data for this paper are available on-line only at http://jmb.or.kr.

## Figures and Tables

**Table 1:** Analysis of variance for the regression equation.

Source	Sum of squares	df	Mean squares	*F*-value	*p*-value
Model	74551.59	9	8283.51	107.40	< 0.0001**
A-Yeast extract	1346.02	1	1346.02	17.45	0.0042**
B-Urea	8652.39	1	8652.39	112.18	< 0.0001**
C-pH	451.79	1	451.79	5.86	0.0461*
AB	0.48	1	0.48	0.01	0.9392†
AC	5565.63	1	5565.63	72.16	< 0.0001**
BC	301.41	1	301.41	3.91	0.0886
A2	7552.10	1	7552.10	97.91	< 0.0001**
B2	19637.99	1	19637.99	254.61	< 0.0001**
C2	25334.82	1	25334.82	328.47	< 0.0001**
Residual	539.90	7	77.13		
Lack of fit	392.94	3	130.98	3.56	0.1256†
Pure error	146.97	4	36.74		
Cor total	75091.49	16			

*: Significant (*p* < 0.05); **: very significant (*p* < 0.01); †: not significant (p > 0.1).

*F: F ratio*; *p-value*: probability value; df: degree of freedom.

R^2^ = 0.9928; adjusted R^2^ = 0.9836; adequate precision = 28.942.

**Table 2:** Effect of various metal ions on the activity of L9^T^ protease.

Metal ions	Final concentration (mM)	Residual activity (± SD, %)
Control	-	100.00 ± 0.79
Li^+^	5	90.19 ± 0.97
K^+^	5	97.71 ± 2.49
Ag^+^	5	104.46 ± 1.27
Mg^2+^	5	97.77 ± 2.00
Ca^2+^	5	104.40 ± 2.12
Mn^2+^	5	96.36 ± 2.13
Fe^2+^	5	84.85 ± 1.25
Co^2+^	5	95.54 ± 0.35
Cu^2+^	5	85.32 ± 1.40
Zn^2+^	5	78.39 ± 2.47
Ba^2+^	5	93.31 ± 2.92
Sr^2+^	5	120.37 ± 3.10
Fe^3+^	5	28.55 ± 3.16
Cr^3+^	5	84.15 ± 1.64

**Table 3:** Effect of different concentrations of organic solvents on L9^T^ protease activity.

Organic solvents	Residual activity (± SD, %)	

	Concentration 5% (v/v)	Concentration 10% (v/v)
Control	100.00 ± 0.10	-
Acetone	88.44 ± 5.31	85.09 ± 3.72
Benzene	95.83 ± 6.36	100.76 ± 2.24
Ethanediol	99.12 ± 2.28	93.37 ± 2.10
Ethanol	90.90 ± 0.77	74.92 ± 4.05
Glycerol	88.76 ± 3.03	99.50 ± 1.22
n-Hexane	91.16 ± 1.91	95.70 ± 3.94
Isopropanol	91.98 ± 1.37	71.64 ± 4.18
Methanol	97.22 ± 3.90	89.77 ± 2.22

**Table 4:** Effect of various surfactants, oxidant, reductants, alkahest and inhibitors on the activity of *O. caprae* L9^T^ protease.

Chemical substances	Working concentration	Residual activity (± SD, %)
Control	-	100.00 ± 3.06
Surfactants		
SDS	10 g/l	23.02 ± 1.18
Tween 20	1% (v/v)	163.41 ± 2.23
Tween 80	1% (v/v)	115.01 ± 1.13
Triton X-100	1% (v/v)	90.34 ± 3.32
Oxidant		
H_2_O_2_	1% (v/v)	88.18 ± 1.18
Reductants		
Dithiothreitol	5 mM	96.96 ± 0.73
β-Mercaptoethanol	5 mM	95.05 ± 2.23
Alkahest		
DMSO	1% (v/v)	110.22 ± 1.70
	5% (v/v)	107.26 ± 0.61
	10% (v/v)	99.94 ± 1.33
Inhibitors		
EDTA	5 mM	30.44 ± 2.77
EGTA	5 mM	49.85 ± 0.60
PMSF	5 mM	0
