# Supplementary material for: Exoproduction and Biochemical Characterization of a Novel Serine Protease from Ornithinibacillus caprae L9T with Hide-Dehairing Activity
Source: J Microbiol Biotechnol. 2021 Nov 20;32(1):99–109. doi: 10.4014/jmb.2108.08037 (PMC9628834; doi:10.4014/jmb.2108.08037)
Supplement: Supplementary file 1 [file jmb-32-1-99-supple.pdf]

Supplementary Material

Exoproduction and biochemical characterization of a novel serine protease from *Ornithinibacillus caprae* L9^T with hide-dehairing activity

Xiaoguang Li[1], Qian Zhang[2], Longzhan Gan[1], Guangyang Jiang[1], Yongqiang Tian[1*], Bi Shi[1*]

[1]Key Laboratory of Leather Chemistry and Engineering, Ministry of Education and College of Biomass Science and Engineering, Sichuan University, Chengdu 610065, PR China

[2]Key Laboratory of Bio-Resources and Eco-Environment, Ministry of Education and College of Life Sciences, Sichuan University, Chengdu 610065, PR China

*Corresponding authors:

Yongqiang Tian

E-mail: yqtian@scu.edu.cn

Address: No.24 South Section 1, Yihuan Road, Chengdu, China

Bi Shi

E-mail: shibi@scu.edu.cn

Address: No.24 South Section 1, Yihuan Road, Chengdu, China

Methods

Selection of medium components

TSB (15 g tryptone, 5 g soy peptone, 5 g NaCl, 1 L H_2O, pH 7.2) was selected as the basal medium to optimize the production of extracellular protease from *Ornithinibacillus caprae* L9^T. Firstly, protease production was carried out in 250 mL Erlenmeyer flasks to evaluate the influence of varying NaCl concentrations (0, 10, 30, 50, 80, 100, 130, 150 and 170 g/L) while keeping other factors constant. Thenceforth, tryptone was replaced by glucose, sucrose, lactose, maltose, fructose, glycerol, soluble starch and yeast extract for screening carbon source. The concentration of optimal carbon source on protease production was studied with a range of 0–30 g/L. Similarly, organic nitrogen sources such as soy peptone, tryptone, peptone, beef extract, casein peptone as well as inorganic nitrogen sources including ammonium sulfate, potassium nitrate and urea were separately supplemented in the fermentation medium for extracellular protease production. And the nitrogen source with the most positive effect was optimized by varying of concentration from 0 to 20 g/L (at intervals of 5 g/L).

Optimization of fermentation conditions

To investigate the effect of incubation time, samples were withdrawn aseptically and monitored for caseinolytic activity at 10 different points in time: 24, 36, 48, 60, 72, 84, 96, 108, 120 h. Individually, the initial fermentation pH was adjusted to the range 5–10 (at intervals of 0.5 unit) with HCl or NaOH. And the effect of incubation temperature on protease production was measured with a range of 25 to 45 ^oC under previously optimized conditions.

Results and discussion

Statistical optimization of protease production

Selection of medium compositions

Generally, the lower yield of protease will bring huge obstacles to subsequent studies, including electrophoresis analyses, biochemical characterization and industrial applications. Thus, the fermentation parameters were optimized using the one factor at-a-time approach to allow the protease hyperproduction. In these studies, the secretion of protease was monitored by measuring the protease activity of the cell-free supernatant. As shown in Fig. S1, strain L9^T was able to secrete protease at a wide range of salinities varying from 30 to 150 g/L NaCl. The protease activity progressively increased with the increase of salt, reached a maximal value at 130 g/L NaCl, and then decreased with 150 g/L NaCl. The results are consistent with other reports, *Bacillus subtilis* BLK-1.5 [1] and *Halobacterium* sp. HP25 [2] showed maximum protease yield in the presence of 70 g/L and 250 g/L NaCl, respectively. This phenomenon might be attributed to the fact that these halophilic/halotolerant microbes could alter the polar lipid composition of cell membranes with increase in salt concentration, thereby further regulating the growth rate and enzyme production [3].

The study of protease production was investigated by culturing strain L9^T in high-salt TSB medium containing different saccharides and organic carbon sources, the results showed that glucose, sucrose, lactose, maltose, fructose and glycerol repressed production of the enzyme when compared with the control (Fig. S2a). This is probably due to the fact that saccharides could act as catabolic repressor or the synthesis of protease is inhibited when the energy status of the cell is high at the tested sugar concentration [4, 5]. Protease production by strain L9^T in the media supplemented with soluble starch or yeast extract was significant enhanced. Many investigators [6, 7] have reported that yeast extract and starch are superior to other substrates in producing proteases. In view of the operability

of yeast extract, and it has been well documented as good carbon source for protease production from halophilic microorganisms [8], yeast extract was selected as the best carbon source and the optimal concentration was determined to be 15 g/L in present study. The results for the screening of nitrogen source showed that urea notablely increased the protease production with the concentration of 5 g/L (Fig. S2b). Similarly, Wang et al. [9] indicated urea could effectively promote the protease production by *Aspergillus oryzae*. Perhaps, as a quick-acting nitrogen source, urea can be directly absorbed and used by bacteria, which is beneficial to the early growth and enzyme production of microorganism.

Optimization of fermentation conditions

The study strain was able to grow and secrete the extracellular protease over a wide range of pH (5–10). The protease production was optimum at pH 9, and decreased drastically with the increasing alkalinity above pH 9 (Fig. S3a). It is speculated that the weak alkaline environment is more conducive for some nutrients and the protease to across the cell membrane [3]. Usually, incubation temperature is an important physical factor, and greatly affects the metabolic transactions of growing cells and protein synthesis. The optimal temperature for protease production was 37 ^{o}C (Fig. S3b), and the optimum fermentation time was concluded to be 72 h (Fig. S1).

References

1. Ali N, Ullah N, Qasim M, Rahman H, Khan SN, Sadiq A, Adnan M. 2016. Molecular characterization and growth optimization of halo-tolerant protease producing *Bacillus Subtilis* strain BLK-1.5 isolated from salt mines of Karak, Pakistan. *Extremophiles* **20:** 395–402.
2. Elbanna K, Ibrahim IM, Revol-Junelles AM. 2015. Purification and characterization of halo-alkali-thermophilic protease from *Halobacterium* sp strain HP25 isolated from raw salt, Lake Qarun, Fayoum, Egypt. *Extremophiles* **19:** 763–774.
3. Mokashe N, Chaudhari B, Patil U. 2018. Operative utility of salt-stable proteases of halophilic and halotolerant bacteria in the biotechnology sector. *Int. J. Biol. Macromol.* **117:** 493–522.
4. Sharma KM, Kumar R, Panwar S, Kumar A. 2017. Microbial alkaline proteases: optimization of production parameters and their properties. *J. Genet. Eng. Biotechnol.* **15:** 115–126.
5. Zambare V, Nilegaonkar S, Kanekar P. 2011. A novel extracellular protease from *Pseudomonas aeruginosa* MCM B-327: enzyme production and its partial characterization. *N. Biotechnol.* **28:** 173–181.
6. Khosravi-Darani K, Falahatpishe HR, Jalali M. 2008. Alkaline protease production on date waste by an alkalophilic *Bacillus* sp. 2-5 isolated from soil. *Afr. J. Biotechnol.* **7:** 1536–1542.
7. Prakasham RS, Rao Ch S, Sarma PN. 2006. Green gram husk——an inexpensive substrate for alkaline protease production by *Bacillus* sp. in solid-state fermentation. *Bioresour. Technol*. **97:** 1449–1454.
8. D'Alessandro CP, De Castro RE, Gimenez MI, Paggi RA. 2007. Effect of nutritional conditions on extracellular protease production by the haloalkaliphilic archaeon

Natrialba magadii. *Lett. Appl. Microbiol.* **44:** 637–642.

9. Wang R, Law RCS, Webb C. 2005. Protease production and conidiation by *Aspergillus oryzae* in flour fermentation. *Process Biochem.* **40:** 217–227.

Table S1. Response surface experimental design factors and levels.

Factor	Units	Symbol	Range of Levels		
			-1	0	1
Yeast extract	g/L	A	10	15	20
Urea	g/L	B	0	5	10
pH		C	8.5	9	9.5

Table S2. The Box-Behnken design of RSM for optimization of the protease production by *O. caprae* L9^T.

Laboratory No.	A: Yeast extract	B: Urea	C: pH	Proteolytic activity (±SD, U/ml)
1	1	-1	0	157.58 ±1.50
2	0	0	0	240.32 ±1.82
3	-1	-1	0	181.19 ±1.19
4	0	-1	-1	124.25 ±2.25
5	1	0	1	60.16 ±8.59
6	1	1	0	90.52 ±4.47
7	0	1	-1	77.82 ±2.09
8	-1	0	1	163.73 ±3.59
9	0	1	1	58.97 ±6.11
10	-1	0	-1	117.70 ±4.39
11	-1	1	0	112.74 ±0.60
12	0	0	0	253.61 ±17.35
13	0	-1	1	140.12 ±3.31
14	0	0	0	250.44 ±12.03
15	0	0	0	239.92 ±5.66
16	1	0	-1	163.33 ±8.18
17	0	0	0	246.47 ±3.59

Table S3. The enzyme gene information contained in the genome of strain L9^{T}.

Gene number	Genbank ID	Length (bp)	Classification
GM000131	WP_155666129.1	1086	Metalloprotease
GM000360	WP_155666531.1	1560	Serine protease
GM000581	WP_155666969.1	3048	Serine protease
GM000845	WP_155667379.1	1719	Serine protease
GM000903	WP_155667464.1	1656	Metalloprotease
GM000927	WP_155667501.1	1389	Glycosidase
GM001202	WP_155667857.1	573	Lipase
GM001373	WP_155668056.1	873	Metalloprotease
GM001495	WP_155668151.1	1224	Serine protease
GM001570	WP_155668210.1	1002	Serine protease
GM001597	WP_155668235.1	1329	Serine protease
GM001857	WP_155668482.1	2337	Glycosidase
GM002151	WP_155668774.1	825	Serine protease
GM002244	WP_155668851.1	1689	Glycosidase
GM002341	WP_155668943.1	1695	Serine protease
GM002381	WP_155668977.1	1776	Glycosidase
GM002590	WP_155669172.1	747	Lipase
GM002605	WP_155669184.1	1095	Metalloprotease
GM003066	WP_155669712.1	2199	Serine protease
GM003134	WP_155669841.1	768	Serine protease
GM003140	WP_155669848.1	735	Lipase
GM003296	WP_155670130.1	1272	Metalloprotease
GM003457	WP_155670438.1	1059	Metalloprotease
GM003551	WP_155670631.1	1476	Amylase
GM003675	WP_155670873.1	1146	Serine protease

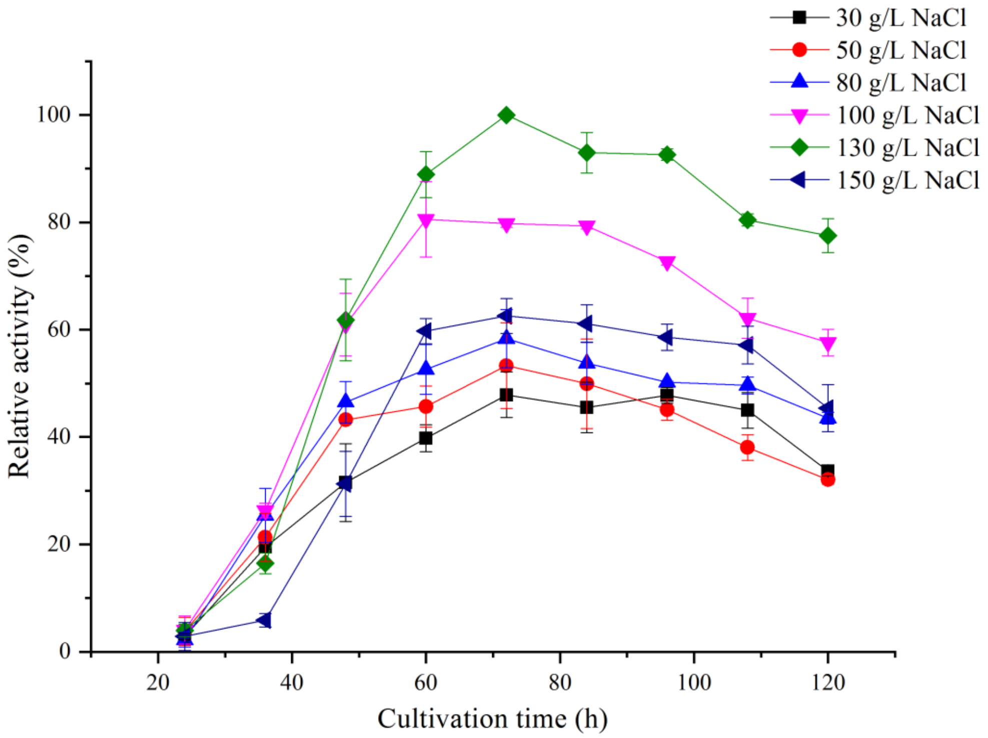

Fig. S1. Effect of fermentation period and salt concentration on the protease production of *Ornithinibacillus caprae* L9^T. Values are represented as mean ± standard deviation of triplicates.

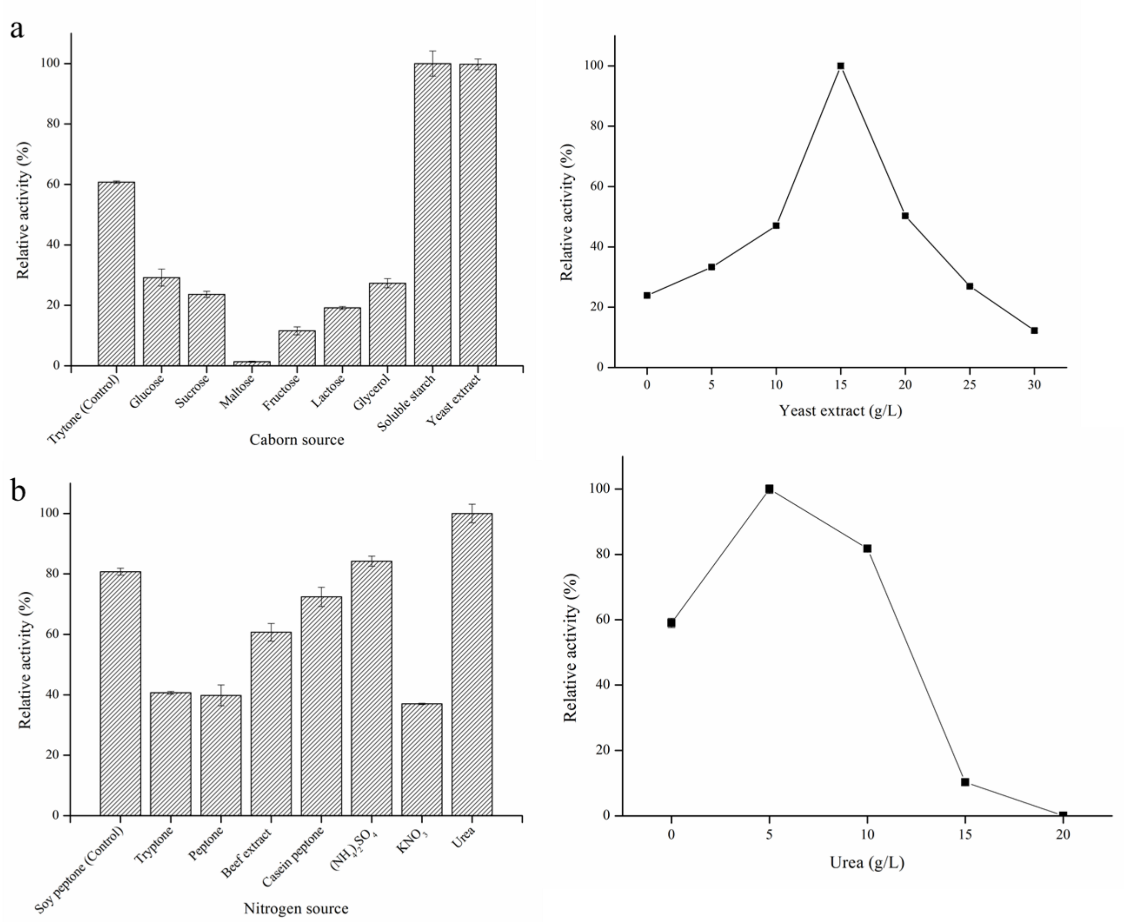

Fig. S2. Effect of nutritional parameters (a, carbon sources; b, nitrogen sources) on protease production from *O. caprae* L9^{T}. Values are represented as mean ± standard deviation of triplicates.

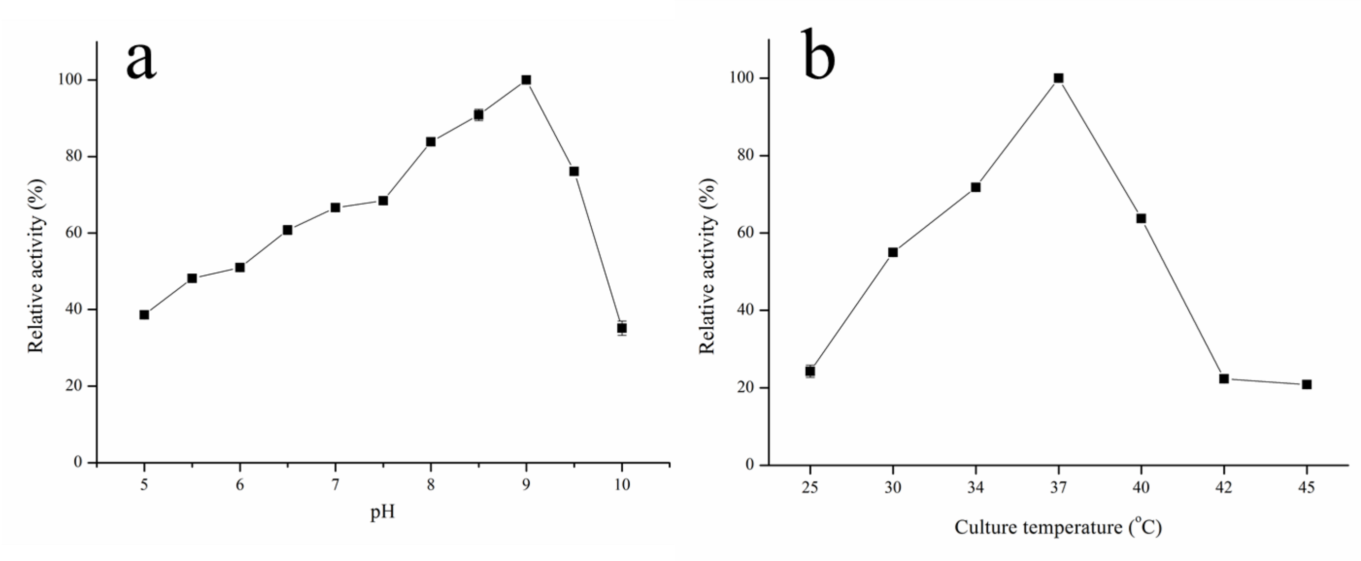

Fig. S3. Effect of fermentation pH (a) and culture temperature (b) on the protease secretion of strain *O. caprae* L9^T. Values are represented as mean ± standard deviation of triplicates.

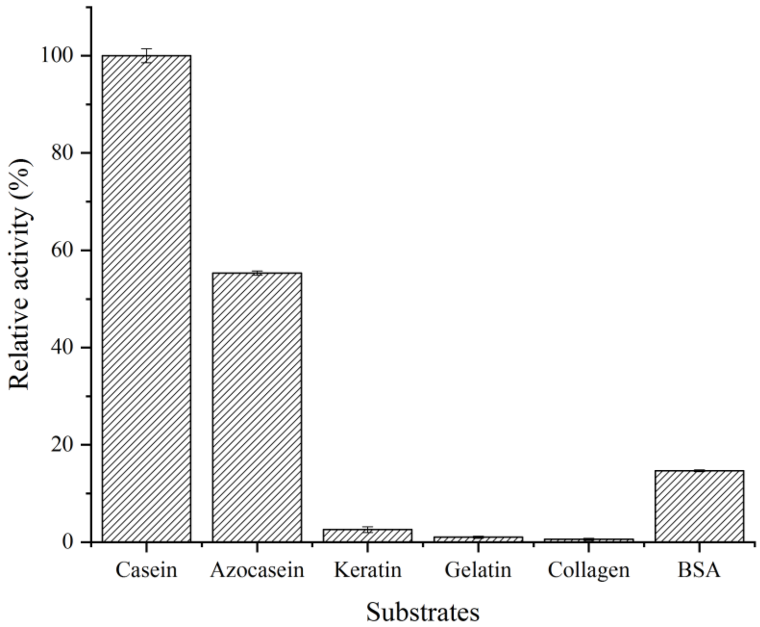

Fig. S4. Effect of substrate specificity on L9^{T} protease activity. Enzymatic activities were determined on each substrate according to standard conditions.

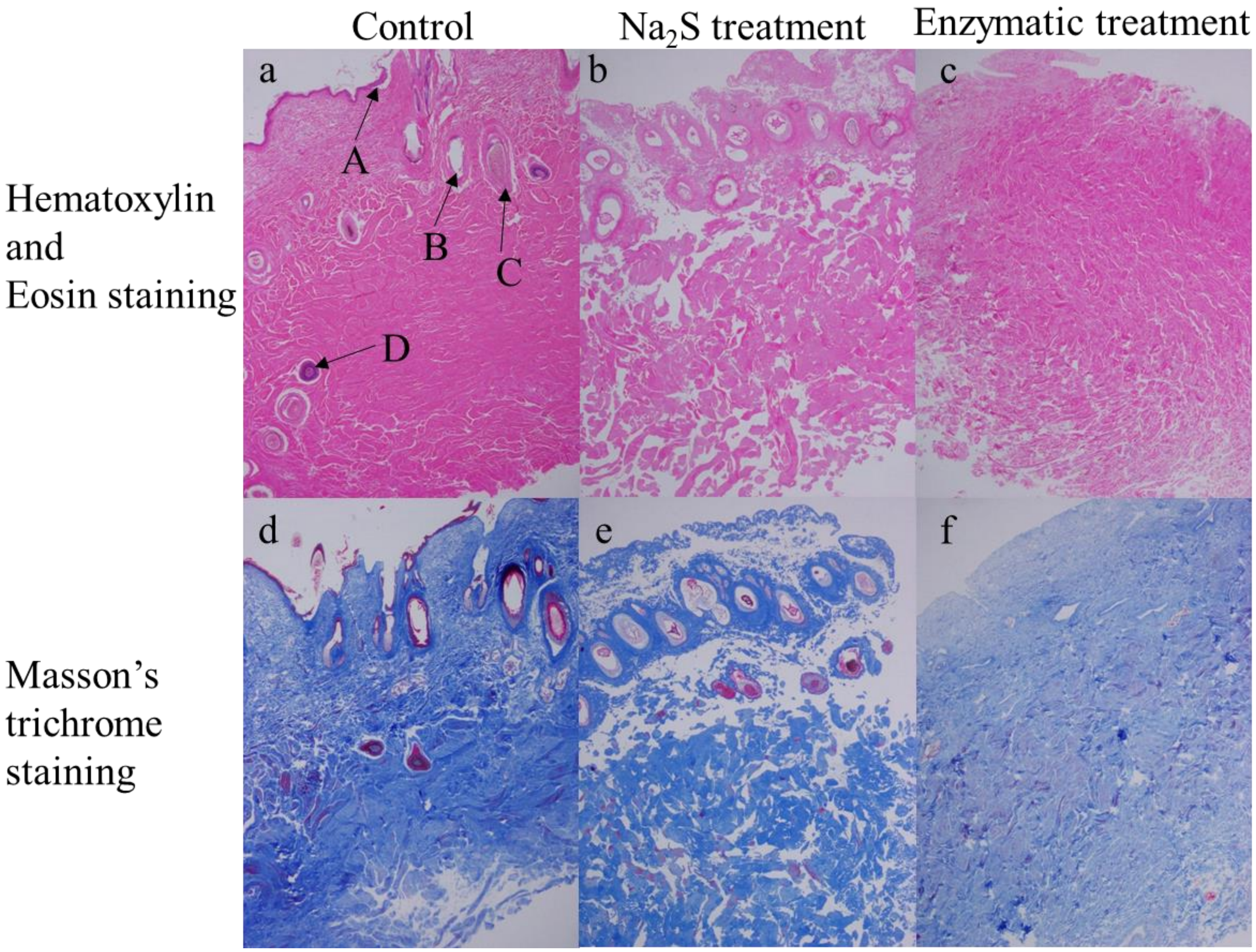

Fig. S5. Images of histology sections of dehaired goatskins in 40× stained with hematoxylin and eosin and Masson's trichrome staining. A: epidermis; B: hair pore; C: hair follicle; D: hair shaft.
